# Modifications in the Intestinal Functionality, Morphology and Microbiome Following Intra-Amniotic Administration (*Gallus gallus*) of Grape (*Vitis vinifera*) Stilbenes (Resveratrol and Pterostilbene)

**DOI:** 10.3390/nu13093247

**Published:** 2021-09-18

**Authors:** Mariana Juste Contin Gomes, Nikolai Kolba, Nikita Agarwal, Dean Kim, Adi Eshel, Omry Koren, Elad Tako

**Affiliations:** 1 Department of Food Science, Cornell University, Stocking Hall, Ithaca, NY 14853, USA; mj395@cornell.edu (M.J.C.G.); nk598@cornell.edu (N.K.); na494@cornell.edu (N.A.); 2 Department of Chemistry and Chemical Biology, Cornell University, Ithaca, NY 14853, USA; dk446@cornell.edu; 3 Azrieli Faculty of Medicine, Bar-Ilan University, Safed 1311502, Israel; adizimer@gmail.com (A.E.); omry.koren@biu.ac.il (O.K.)

**Keywords:** resveratrol, pterostilbene, stilbene, polyphenol, intra-amniotic administration, gut microbiome, brush border functionality

## Abstract

This efficacy trial evaluated the effects of two polyphenolic stilbenes, resveratrol and pterostilbene, mostly found in grapes, on the brush border membrane functionality, morphology and gut microbiome. This study applied the validated *Gallus gallus* intra-amniotic approach to investigate the effects of stilbene administration versus the controls. Three treatment groups (5% resveratrol; 5% pterostilbene; and synergistic: 4.75% resveratrol and 0.25% pterostilbene) and three controls (18 MΩ H_2_O; no injection; 5% inulin) were employed. We observed beneficial morphological changes, specifically an increase in the villus length, diameter, depth of crypts and goblet cell diameter in the pterostilbene and synergistic groups, with concomitant increases in the serum iron and zinc concentrations. Further, the alterations in gene expression of the mineral metabolism proteins and pro-inflammatory cytokines indicate a potential improvement in gut health and mineral bioavailability. The cecal microbiota was analyzed using 16S rRNA sequencing. A lower α-diversity was observed in the synergistic group compared with the other treatment groups. However, beneficial compositional and functional alterations in the gut microbiome were detected. Several key microbial metabolic pathways were differentially enriched in the pterostilbene treatment group. These observations demonstrate a significant bacterial–host interaction that contributed to enhancements in intestinal functionality, morphology and physiological status. Our data demonstrate a novel understanding of the nutritional benefits of dietary stilbenes and their effects on intestinal functionality, morphology and gut microbiota in vivo.

## 1. Introduction

Polyphenols are a class of bioactive compounds of plant origin that may play an important part in reducing the risk of chronic diseases [1]. Polyphenols are naturally produced by plants as secondary metabolites and are generally involved in plant defense pathways. Recently, there has been growing scientific attention for the health benefits of dietary polyphenols. Epidemiological studies on polyphenol consumption have yielded promising evidence for polyphenols possessing health-promoting properties. These include protection against cardiovascular diseases and diabetes, likely secondary to antioxidant and anti-aging properties [2]. In a previous epidemiological study that focused on grape-originating polyphenols, researchers devised the expression “the French Paradox” to describe the observed lower coronary heart disease (CHD) mortality in French people despite their high saturated fat intake [3]. It has been postulated that moderate wine consumption provides a significant amount of dietary polyphenols, thus lowering the incidence of CHD, which may explain this dichotomy.

Resveratrol is a stilbene, a polyphenolic subclass primarily found in grapes [4]. Resveratrol has demonstrated chemoprotective, cardioprotective, anti-inflammatory and neuroprotective activities, likely due to its ability to scavenge free radicals and chelate both redox-active and redox-inactive metals [5,6]. Grapes are the most produced fruit crop in the United States. Globally, over 77.8 million tons of grapes are produced annually, with 57% being wine grapes, 36% being table grapes and 7% being dried grapes [7]. Therefore, as grape production and consumption increases, it is imperative to maximize understanding of the specific health effects of bioactive compounds that are found in this fruit. Pterostilbene is another stilbene and an analog of resveratrol present in smaller quantities within grapes and grape products, including wine [8,9]. The two methoxy substituents in pterostilbene increase the lipophilicity of the compound relative to resveratrol, which contains two hydroxy substituents. In the plant, these compounds serve as the phytoalexins found in grape skins and stems [10]. Pterostilbene is more easily absorbed than resveratrol in the gut lumen because it exhibits higher bioavailability (80%, compared with 20% from resveratrol) [11,12].

Although bioactive polyphenols can chelate iron (Fe) and zinc (Zn), studies using various in vitro and in vivo models to evaluate the effects of dietary stilbenes on mineral metabolism are not elucidated [13,14]. This is especially the case with resveratrol and pterostilbene, as there is no substantial research which has evaluated the effects of these compounds on duodenal brush border membrane (BBM) functionality or the gastro intestinal tract microbiota, which play a major role in mineral bioavailability and metabolism [15]. Therefore, by utilizing the *Gallus gallus* in vivo model [16,17,18,19,20,21,22,23], we aim to investigate the effects of the intra-amniotic administration of stilbenes (resveratrol and pterostilbene) on the multiple criterion that influence mineral bioavailability and absorption, the mineral-dependent proteins’ gene expression and BBM morphology. Additionally, the cecal content was utilized for 16S rRNA gene sequencing, and potential alterations in the gut microbiota structure and function related to the administration of stilbenes were assessed. We hypothesize that intra-amniotic administration of resveratrol and pterostilbene, and the combination of both for a synergistic assessment, will lead to increased mineral absorption through favorable alterations in BBM functionality and tissue morphology and beneficially modulate the intestinal microbial populations.

## 2. Materials and Methods

### 2.1. Sample Preparation

Resveratrol (≥99%) and pterostilbene (≥97%) (Sigma-Aldrich, St. Louis, MO, USA) were used for the intra-amniotic administration experiment. In addition, three grape (*Vitis vinifera*) variety samples (Cabernet Franc, Concord, and Noiret) were obtained from the Cornell University vineyards in Lansing and Geneva, NY. The grapes were harvested in bunches. With berries still on the stems and prior to separation, the samples were immediately frozen at −80 °C. Before the analysis, the whole grapes were crushed, lyophilized and then ground by taking fruit from each bundle evenly.

### 2.2. Polyphenols Analysis

#### 2.2.1. Grape Sample Preparation

Each ground whole grapes sample (0.5 g) was added to 5 mL of methanol:H_2_O (50:50 *v*/*v*). The mixture was shaken for 1 min and incubated in a water bath at 24 °C for 20 min, after which the samples were vortexed, kept on Rocker (Labnet Int., Inc., Edison, NJ, USA) for 60 min, and centrifugated (4000× *g* for 15 min). The supernatants were removed, filtered (0.2 μm Teflon™ syringe filter) and kept at −20 °C [24,25,26].

#### 2.2.2. Liquid Chromatography-Mass Spectrometry (LC-MS) Analysis

The sample extracts and polyphenol standards were diluted to 30% methanol for injection, and the compounds were separated with an ACQUITY BEH C18 column (2.1 × 100 mm, 1.7 µm) at 50 °C (Waters, Milford, MA, USA). Two-component and two-segment gradient elution were employed. The two components of the gradient were composed of 0.1% aqueous formic acid (FA) as Eluent A and 0.1% FA in methanol as Eluent B. The flow rate was 0.5 mL/min. The initial conditions were 5% Eluent B. The first gradient segment was linear over 3.5 min from 5% to 30% Eluent B, followed by a second linear segment from 30% to 60% Eluent B over 5.5 min. The column was rinsed at 90% Eluent B for 0.5 min and re-equilibrated at 5% Eluent B prior to the next injection. The column was fixed to a photodiode array detector (PDA) (Waters, Milford, MA, USA) to measure the UV absorbance peaks in the standards and grape samples in the range of 225–498 nm. Flow was then directed into a mass sensitive detector (QDa, Waters, Milford, MA, USA), and it was used to generate extracted ion chromatograms at the subject m/z values. The ionization parameters were set as follows: a probe temperature of 550 °C and cone voltage +/− 6V. The mass range investigated was 100–250 Da. The extracts were run in positive ion modes. The compounds were identified by characteristic retention times and mass spectra and compared to the standards. LC and CMS instrumentation and result analyses acquisition were monitored via Empower^TM^ software. Individual polyphenols were identified through comparison of the m/z and LC retention timing with the standards. The MS intensity peaks from seven replications were used for creating polyphenol standard curves for stilbenes.

### 2.3. Animals and Study Design

Cornish cross-fertile broiler eggs (*n* = 72) were purchased from a hatchery (Moyer’s Chicks, Quakertown, PA, USA). The eggs were incubated in ideal conditions at the Cornell University Animal Science poultry farm incubator. All animal protocols were approved by the Cornell University IACUC (protocol code: 2020-0077). 

#### Intra-Amniotic Administration

The pure resveratrol and pterostilbene were individually diluted in 18 MΩ H_2_O. The intra-amniotic administration followed the methodology previously described [17,23,24,25,26,27]. On the 17th day of embryonic development, the eggs with viable embryos (*n* = 60) were randomly allocated into 6 groups (*n* = 10) with a similar weight frequency distribution. For the intra-amniotic treatment, 1 mL of solution per egg was injected into the amniotic fluid (via candling) with the aid of a 21-gauge needle. After the procedure, the injection sites were sealed with cellophane tape, and the eggs were distributed into six groups: (1) no injection; (2) 18 MΩ H_2_O; (3) 5% inulin; (4) 5% resveratrol; (5) 5% pterostilbene; and (6) synergistic: 4.75% resveratrol and 0.25% pterostilbene. As was included in previous studies [24,25,26,27], inulin was used as a positive control, as it belongs to a class of polysaccharides that have been shown to beneficially modulate the gut microbiome when consumed. The eggs were equally distributed in each incubator location to reduce possible allocation bias. Upon hatching (21 days), the hatchlings were euthanized by CO_2_ exposure, and the blood, liver, heart, pectoral muscle, small intestine and cecum were obtained.

### 2.4. Blood Analysis and Hemoglobin Measurements

Blood was collected using micro-hematocrit heparin-coated capillary tubes (Fisher Scientific, Waltham, MA, USA). The hemoglobin (Hb) concentration was assessed spectrophotometrically using the QuantiChrom^TM^ Hb Assay (DIHB-250, BioAssay Systems, Hayward, CA, USA) and according to specific instructions.

### 2.5. Fe and Zn Content in the Serum and Liver Samples

The serum (50 µL) and liver (0.5 g) samples were mixed with 3.0 mL of a 60:40 (*v/v*) solution of nitric acid (HNO_3_) and perchloric acid (HClO_4_) and incubated overnight. After this, the samples were heated for 2 hours at 120 °C and added to 0.25 mL of 40 µg/g yttrium (internal standard), and the block temperature was increased to 145 °C for 2 h and then 190 °C for 10 min. At the end of these procedures, the samples were cooled, diluted to a total volume of 20 mL, mixed and transferred into autosampler tubes for analysis (inductively coupled plasma atomic emission spectrometer) [26,28] (Thermo, Franklin, MA, USA).

### 2.6. Isolation of the Total RNA from the Duodenum and Liver Tissue Samples

The RNA was isolated as described previously [25,26,27,29] (RNeasy Mini Kit, Qiagen Inc., Valencia, CA, USA), and 30 mg of the duodenal tissue or liver tissue (*n* = 8) was used to extract the total RNA. The tissues were homogenized in buffer RLT^®^ with β-mercaptoethanol (rotor-stator homogenizer). After this, the samples were centrifuged for 3 min at 8000× *g*, and an aliquot of the supernatant was added with 1 volume of 70% ethanol. Then, 700 μL of this solution was added to a mini column and centrifuged for 15 s at 8000× *g*. In new 2-mL collection tubes, the RNeasy columns of 0.5 mL of buffer RPE^®^ were added, centrifugated for 15 s at 8000× *g*, and the buffer RPE^®^ was added once more, followed by centrifugation for 2 min at 8000× *g*. Finally, the RNA was eluted in 50 μL of RNase-free water and quantified by absorbance at 260 and 280 nm.

The integrity of the 18S ribosomal RNAs was tested (1.5% agarose gel electrophoresis followed by ethidium bromide staining). DNA contamination was addressed by using a TURBO DNase removal kit (AMBION, Austin, TX, USA). The procedures were conducted in RNase-free conditions.

### 2.7. Real-Time Polymerase Chain Reaction (RT-PCR) and Primer Design

RT-PCR was conducted as described [24,25,26]. The cDNA was created from a 20-µL reverse transcriptase (RT) reaction (BioRad C1000 Touch Thermocycler) and using the Improm-II Reverse Transcriptase Kit (Promega, Madison, WI, USA). The cDNA content was assessed by absorbance at 260 and 280 nm, with an extinction coefficient of 33 (single-stranded DNA). Genomic DNA contamination was evaluated via a real-time RT-PCR assay for the reference gene samples [17,18,30].

The primers used in the real-time PCR were formulated in accordance with relevant gene sequences (GenBank database), via Real-Time Primer Design Tool software (IDT DNA, Coralville, IA, USA) and as was previously described [24,25,26]. The primer sequences related to iron, zinc, calcium and magnesium metabolism, immune response, hypertension and BBM functionality that were utilized are indicated in Table 1. The specificity of the primers was verified via BLAST search versus the genomic National Center for Biotechnology Information (NCBI) database. The reference gene used was the 18S rRNA specific for the *Gallus gallus* model.

### 2.8. Real-Time qPCR Design

The procedures were performed as previously published [24,25,26,28]. The prepared cDNA was applied in a 10-µL reaction containing 2 × BioRad SSO SYBR Green Supermix (Hercules, CA, USA). Table 1 shows the primers used in this study. The optimal MgCl_2_ concentration achieved, for each reaction, an amplification plot (lowest cycle product, Cp), the uppermost fluorescence intensity and the steepest amplification slope. No template control of nuclease-free water was used in order to eliminate DNA contamination. For each reaction (duplicates), 8 µL of the master mix and 2 µL cDNA were pipetted into a 96-well plate, and for the standard curve, seven points were evaluated in duplicate. The Bio-Rad CFX96 Touch (Hercules, CA, USA) system was used to amplify the double-stranded DNA under optimal PCR conditions: denaturing at 95 °C for 30 s, 40 cycles of denaturing at 95 °C for 15 s, changing annealing temperatures as indicated by Integrated DNA Technologies for 30 s and elongating at 60 °C for 30 s.

According to Bio-Rad CFX Maestro 1.1 (Version 4.1.2433.1219, Hercules, CA, USA), the Cp values based on the “second derivative maximum” were used to obtain data on the expression levels of the genes. The assays were calculated by including a standard curve in the real-time qPCR analysis, and a standard curve with four points was prepared by a 1:10 dilution (duplicatea). A graph of the Cp vs. log 10 concentrations was produced, and the efficiencies were calculated as 10 (1/slope). The specificities of the amplified real-time RT-PCR products were confirmed by melting curve analysis (60–95 °C) after 40 cycles, resulting in several different specific products with specific melting temperatures.

### 2.9. 16S rRNA Gene Amplification and Sequencing

As was formerly described [22], cecal samples were used to extract microbial genomic DNA (PowerSoil DNA isolation kit, MoBio Laboratories Ltd., Carlsbad, CA, USA). The V4 hypervariable region of the bacterial 16S rRNA gene was PCR-amplified per sample (515F-806R primers) and with 12-base barcodes [31]. Each PCR reaction was composed of 25 µL Primestar max PCR mix (Takara Kusatsu, Shiga, Japan), 2 µM of each primer, 17 µL of ultra-pure water and a 4-µL DNA template, as indicated: a denaturing step for 3 min at 95 °C, 30 cycles of 10 s at 98 °C, 5 s at 55 °C, 20 s at 72 °C and final elongation at 72 °C for 1 min. The PCR products were purified (Beckman Coulter, Atlanta, GA, USA) and quantified (Quant-iT PicoGreen dsDNA quantitation kit, Invitrogen, Carlsbad, CA, USA). Equimolar ratios of the samples were pooled and sequenced (Illumina, Inc., Madison, WI, USA).

#### 16S rRNA Gene Sequence Analysis

The 16S rRNA gene sequence analysis was performed using QIIME2 [32] as previously described [28]. The sequence reads were demultiplexed by per-sample barcodes, and the Divisive Amplicon Denoising Algorithm (DADA2) [33] was used to correct the Illumina-sequenced amplicon read errors. Sequences from a phylogenetic tree were taxonomically classified using the Greengenes [34] reference database at a confidence threshold of 99%. The Greengenes taxonomies were used to generate summaries of the taxonomic distributions of the features across different levels (phylum, order, family and genus). Low-abundance amplicon sequence variants (ASVs) (observed only in one sample per group) were filtered out from the ASV table. The alpha and β diversity were calculated from samples containing at least 9.800 sequences. The alpha diversity parameter, a measure of the richness of the bacterial community, was calculated using the Shannon Index, and the beta diversity was assessed by a Jaccard similarity distance matrix. Linear discriminant analysis effect size (LEfSe) [35] was conducted according to the relative abundances of each bacterial community to verify the highlights that significantly differed between the samples. Metagenome functional predictive analysis of the communities was performed via phylogenetic investigation in PICRUSt [36] software (version 1.1.3). The identified sequences’ abundances were normalized by the 16S rRNA gene copy number and compared to a known phylogenetic reference tree (Greengenes database). The enriched metabolic pathways were assessed via the Kyoto Encyclopedia of Genes and Genomes (KEGG). Data indicating significant differences in the functional pathways between the groups was plotted.

### 2.10. Morphometric Examination of Duodenal Tissue

Villus epithelium analysis was conducted as previously published [29,37]. The duodenal samples were soaked in buffered formaldehyde (4% (*v*/*v*)), dehydrated, cleared and embedded in paraffin. Numerous sections were cut with a thickness of 5 µm and put on slides. The sections were deparaffinized in xylene, after which they were rehydrated in a series of graded alcohol. Finally, the slides were stained with Alcian Blue–periodic acid-Schiff and investigated under a light microscopy. The variables were assessed (light microscope, EPIX XCAP software, standard version, Olympus, Waltham, MA, USA) for the following: villus length, villus diameter, depth of crypts, goblet cell diameter, crypt goblet cell number and villus goblet cells type number (acidic, neutral or mixed). Four segments for each biological sample and five biological samples per treatment group were examined. The goblet cells were enumerated at 10 villi/sample, and the means were calculated for statistical analysis.

### 2.11. Statistical Analysis

The values in this paper were expressed as means and standard errors of the means (SEM). The experimental groups for the intra-amniotic assay were in a completely randomized order. The results were analyzed by Analysis of Variance (ANOVA). Following this, to obtain the “*p*-value”, a post hoc Duncan test was utilized to compare all groups. SPSS software version 26.0 was used to carry out the statistical analysis, where *p* < 0.05 was established as the level of significance. For the microbiome results, the Shannon Index alpha diversity metric was utilized to determine the bacterial richness in the samples, and the differences among the groups were analyzed by ANOVA. For assessing β diversity, the Jaccard distances were calculated by the pairwise PERMANOVA test. Statistically significant *p*-values linked to microbial functions were determined by LEfSe and corrected for multiple comparisons according to the Benjamini–Hochberg false discovery rate (FDR). SAS version 9.3 (SAS Institute, Cary, NC, USA) was used for the statistics.

## 3. Results

### 3.1. Stilbenes Compounds Identified in Grape Samples

The resveratrol, the compound used in the in ovo experiment, was shown to be found in commonly grown *Vitis* spp. grapes from the central New York region. However, the pterostilbene was not detected in the grape samples evaluated, and the retention time is presented in Table 2.

### 3.2. In Ovo Assay

#### 3.2.1. Blood hemoglobin (Hb), Serum and Hepatic Fe and Zn Concentrations

The Hb concentration for the synergistic treatment group was significantly lower (*p* < 0.05) compared with the controls and other treatment groups. Furthermore, there was no difference between the 5% resveratrol and 5% pterostilbene groups compared with the control groups (Table 3).

The synergistic group presented higher Fe and Zn levels in the liver and in the serum compared with the no injection group (*p* < 0.05). The 5% pterostilbene group had a significantly greater serum Zn concentration compared with the control groups and 5% resveratrol group (*p* < 0.05) and presented no difference compared with the synergistic group (*p* > 0.05). 

#### 3.2.2. Gene Expression of BBM Proteins, BBM Functionality, the Immune System and Hypertension

**Figure 1:**
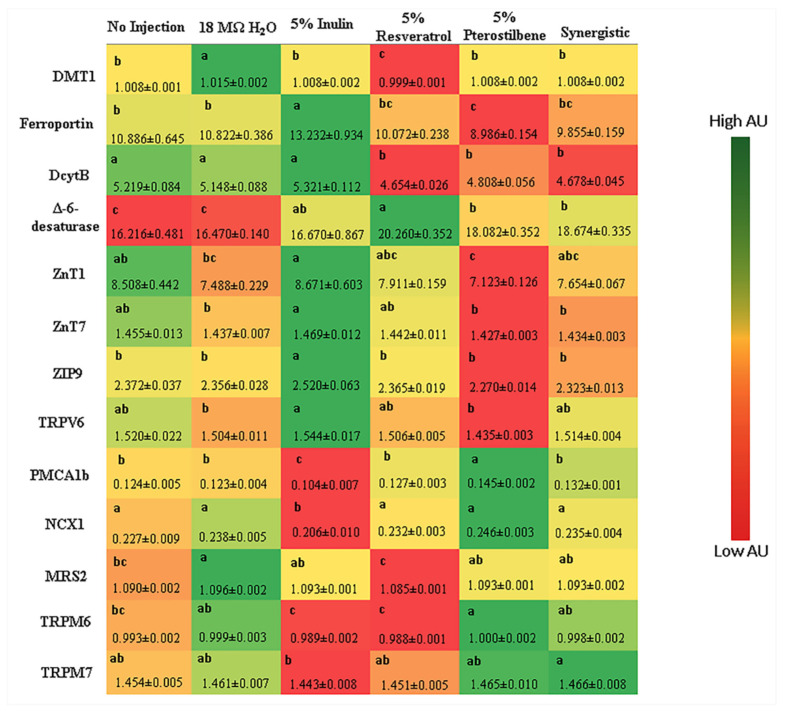
Effect of stilbenes administration on intestinal gene expression of proteins involved with mineral metabolism. Values are the means ± SEM (*n* = 8). ^a–c^ Per gene, treatment groups not indicated by the same letter are significantly different (*p* < 0.05). Iron metabolism: DMT1 = divalent metal transporter 1; DcytB = duodenal cytochrome b. Zinc metabolism: Znt and ZIP = zinc transporter proteins. Calcium metabolism: TRPV6 = transient receptor potential cation channel subfamily V member 6; PMCA1b = plasma membrane Ca^2+^ ATPase isoform 1b; NCX1 = sodium-calcium exchanger 1. Magnesium metabolism: MRS2 = mitochondrial magnesium channel type 2; TRPM6 and TRPM7 = transient receptor potential melastatin.

Figure 1 shows the gene expression of various proteins indirectly involved in mineral metabolism. The expression of DcytB was significantly downregulated in all treatment groups compared with the control groups (*p* < 0.05), and the relative expression of ferroportin was downregulated in the treatment groups compared with the 5% inulin control (*p* < 0.05). DMT1 relative expression was the lowest in the 5% resveratrol group compared with the other treatments and the controls (*p* < 0.05). The Δ-6-desaturase is involved with the fatty acid biosynthesis and is an indicator of Zn deficiency [38,39,40]. Its activity was significantly upregulated in all treatment groups compared with the no injection and 18 MΩ H_2_O control groups (*p* < 0.05), and in the 5% pterostilbene and synergistic groups, the Δ-6-desaturase gene expression was similar to the 5% inulin control (*p* > 0.05).

The relative expressions of the Zn transporters and importers (ZnT1, ZnT7 and ZIP9) presented no difference (*p* > 0.05) compared with the no injection and 18 MΩ H_2_O control groups. Related to mineral absorption and the functionality of BBM proteins, after the administration of grape stilbenes, we observed an upregulation in proteins related to calcium (PMCA1b and NCX1) and magnesium metabolism (TRPM6) in the 5% pterostilbene and synergistic groups compared with the 5% inulin control group (*p* < 0.05) but with no difference related to the other control groups (*p* > 0.05). In addition, we did not observe differences in the relative expressions of TRPV6, MRS2 or TRPM7 in the treatment groups compared with the no injection and 18 MΩ H_2_O controls (*p* > 0.05) (Figure 1).

**Figure 2:**
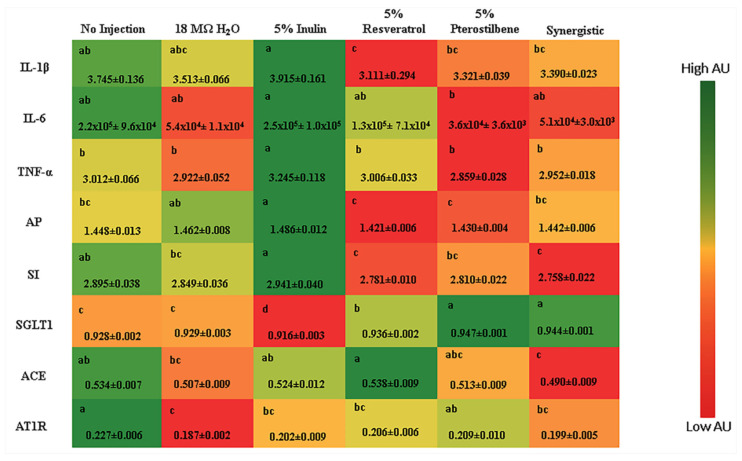
Intestinal gene expression of proteins involved with the immune system, BBM functionality and hypertension. Values are the means ± SEM (*n* = 8). ^a–c^ Per gene, treatment groups not indicated by the same letter are significantly different (*p* < 0.05). IL-1β: interleukin 1β; IL-6: interleukin-6; TNF-α: tumor necrosis factor alpha; AP: aminopeptidase; SGLT1: sodium-glucose transport protein 1; SI: sucrose isomaltase; ACE: angiotensin-converting enzyme; AT1R: angiotensin II receptor type I.

The effect of stilbenes administration on BBM proteins involved with inflammation, BBM functionality and hypertension were also evaluated (Figure 2). The inflammatory cytokines IL-1β and TNF-α were downregulated (*p* < 0.05) in all experimental groups (5% resveratrol, 5% pterostilbene and synergistic) compared with the 5% inulin control group. IL-6 was downregulated (*p* < 0.05) in the 5% pterostilbene group compared with the 5% inulin control, but there was no difference between the other control groups (no injection or 18 MΩ H_2_O) (*p* > 0.05). Related to BBM functionality, the relative expression of AP was downregulated in the 5% resveratrol and 5% pterostilbene groups relative to the 18 MΩ H_2_O and 5% inulin control groups (*p* < 0.05), and SI was down regulated in the 5% resveratrol and synergistic groups compared with the no injection and 5% inulin control groups (*p* < 0.05). However, it was interesting that the relative expression of SGLT1 was upregulated in all three experimental groups compared with the controls (*p* < 0.05) (Figure 2).

The gene expression of proteins related to the hypertension process was also evaluated, and the relative expression of ACE was downregulated in the synergistic group compared with the no injection and 5% inulin control groups (*p* < 0.05), and AT1R gene expression was downregulated in the 5% resveratrol and synergistic groups compared with the no injection control (*p* < 0.05) (Figure 2).

#### 3.2.3. Duodenal Morphometric Parameters

The 5% pterostilbene and synergistic treatment groups presented elevated (*p* < 0.05) villus lengths and diameters versus the controls and higher (*p* < 0.05) depths of the crypts relative to the 18 MΩ H_2_O and 5% inulin controls (Table 4).

All the treatment groups demonstrated a higher (*p* < 0.05) goblet cell diameter compared with the controls, and the synergistic group presented the highest (*p* < 0.05) goblet cell diameter relative to all the experimental groups. However, the synergistic treatment group demonstrated a significantly lower (*p* < 0.05) crypt goblet cell number relative to the other treatment groups and the 18 MΩ H_2_O and 5% inulin controls. Furthermore, there was a significantly lower (*p* < 0.05) amount of acidic villi goblet cells in the synergistic group and a significantly higher (*p* < 0.05) amount of mixed villi goblet cells in the synergistic and 5% pterostilbene treatment groups relative to the controls (Table 5).

### 3.3. Analysis of the Gut Microbiota

**Figure 3:**
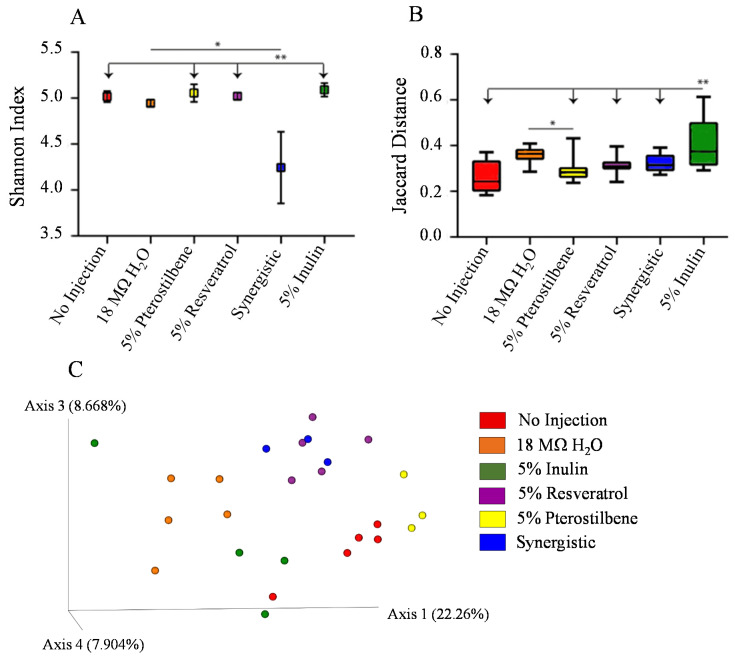
Microbial diversity of the cecal microbiome after the intra-amniotic administration of stilbenes. (**A**) Measure of α diversity using the Shannon Index. (**B**) Boxplot of Jaccard similarity distances within groups, with significant differences between groups (arrows) compared with the 5% inulin group. (**C**) Principal Coordinates Analysis (PCoA) based on the Jaccard similarity distances of cecal microbial communities from the different treatment groups. Each dot represents one animal, and the colors represent the different treatment groups (*n* = 5). * *p* < 0.05. ** *p* < 0.01.

The gut microbial diversity differences among the treatment and control groups are shown in Figure 3. Significantly lower α diversity was identified in the synergistic group relative to the other treatment groups (*p* < 0.05) (Figure 3A). The β diversity indicates the variation in bacterial communities between samples (Figure 3B,C). Spatial ordination of the data based their collections of sequences showed statistical differences in the distance metrics among the 5% inulin control compared with all other experimental groups (*p* < 0.01) and a lower β diversity in the 5% pterostilbene compared with the 18 MΩ H_2_O control (*p* < 0.05). Furthermore, the β diversities of the three treatment groups with stilbenes were not significantly different (*p* > 0.05).

**Figure 4:**
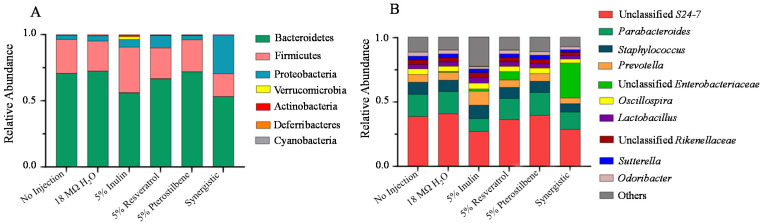
Compositional differences of gut microbiota in response to the intra-amniotic administration of stilbenes. The graph represents the percentage of the relative abundance of a specific phylum in relation to the other phyla identified in the same treatment group. (**A**) Phylum level differences measured upon hatching. (**B**) Genus level differences measured on the day of hatching (day 21).

The predominant phyla in all treatment groups were Bacteroidetes and Firmicutes (Figure 4A). In general, the cecal gut microbiome of *Gallus gallus* at the phylum level is very similar to that observed in the human gut, as has been previously demonstrated in human clinical trials [41]. Proteobacteria, Verrucomicrobia, Actinobacteria, Deferribacteres, and Cyanobacteria were detected in the guts of all the treatment groups in lower abundances, and there was a lower (*p* < 0.05) abundance of Actinobacteria in the synergistic treatment group relative to the 5% inulin control. In addition, there was a tendency to reduce (*p* = 0.083) the abundance of Firmicutes in the synergistic group relative to the 5% inulin control (Figure 4A). At the genus level, the predominant bacteria in all treatment groups was Unclassified S24-7, belonging to a family of bacteria within the Bacteroidetes phyla. However, we did not observe a difference (*p* > 0.05) in the bacteria composition at the genus level (Figure 4B).

**Figure 5:**
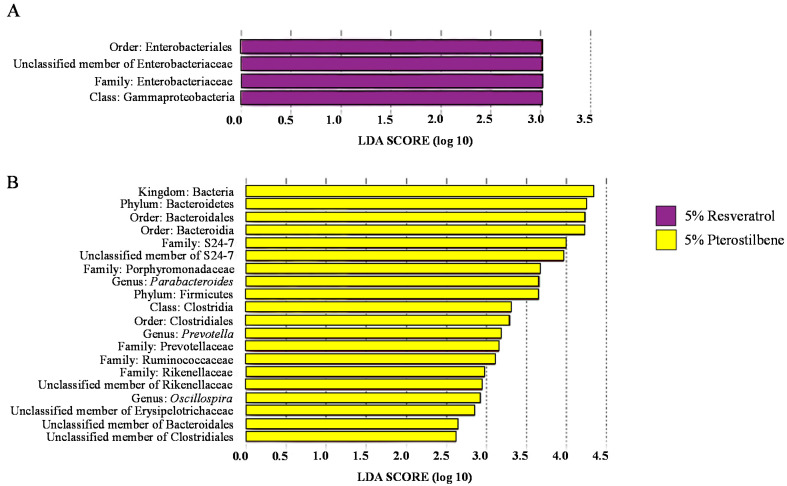
LEfSe analysis identifying the most differentially enriched taxa in the intra-amniotic administration of stilbenes. (**A**) In purple, computed linear discriminant analysis (LDA) scores of the relative abundance difference between the 5% resveratrol group and the no injection control group. (**B**) In yellow, computed LDA scores of the relative abundance difference between the 5% pterostilbene group and the no injection control group.

Furthermore, the linear discriminant analysis effect size method (LEfSe) was used to investigate the bacterial biomarkers and isolate gut microbiome differences between the treatment groups. The Enterobacteriales order and Enterobacteriaceae family, a member of the Proteobacteria phylum, was significantly enriched (*p* < 0.05) in the 5% resveratrol treatment group compared with the no injection control group (Figure 5A). However, in the 5% pterostilbene treatment group, we observed that the most differentially enriched taxa were related to members of the Bacteroidetes phylum, such as the S24-7 family, members of the Clostridiales order and the *Oscillospira* genus compared with the no injection control group (Figure 5B).

**Figure 6:**
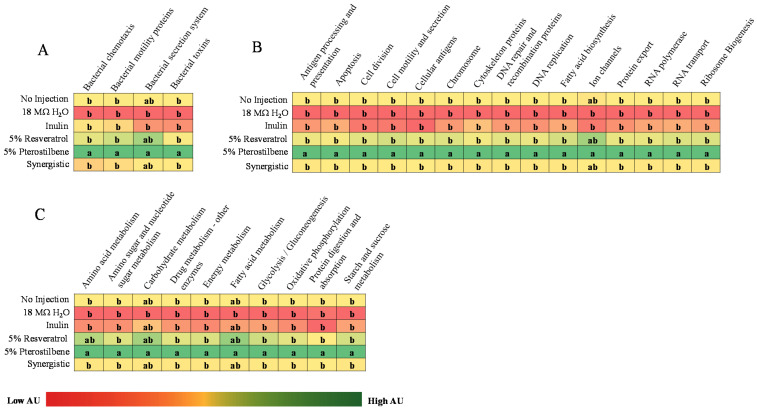
Relative significance of differentially enriched microbial metabolic pathways in cecal microbiota after the intra-amniotic administration of stilbenes. (**A**) Bacterial processes. (**B**) Cellular processes. (**C**) Metabolic processes. ^a–b^ Treatment groups not indicated by the same letter are significantly different by ANOVA (*p* < 0.05).

To investigate any alterations in the metagenomic potential of the gut microbiota, metagenome functional predictive analysis was carried out using PICRUSt software. The feature abundance was normalized by the 16S rRNA gene copy number and identified using the Greengenes database, and the KEGG orthologs prediction was calculated. The 5% pterostilbene group demonstrated significant enrichment (*p* < 0.05) of all the KEGG metabolic pathways identified compared with the no injection, 18 MΩ H_2_O and 5% inulin controls, except by metabolic processes related to the bacterial secretion system, ion channels and carbohydrate and fatty acid metabolism. However, there was no difference (*p* > 0.05) in the KEGG metabolic pathways between the 5% resveratrol and synergistic treatment groups compared with the controls (Figure 6).

## 4. Discussion

Stilbenes, such as resveratrol and pterostilbene, are plant-bioactive compounds primarily found in grapes. These compounds present potent proposed health benefits and may play an essential role in reducing the risk of chronic diseases, protection against cardiovascular diseases and diabetes [42,43,44]. In the plant, stilbenes serve as phytoalexins, which are defensive substances produced in response to infections [45]. Previous studies, which evaluated the benefits of resveratrol and pterostilbene, have posited chemoprotective [46], cardioprotective and anti-inflammatory activities [42,43,44,47]. However, limited research that assessed the effects of resveratrol and pterostilbene on the intestinal microbiome in vivo [48,49] is available. In addition, nothing in the literature presently exists which directly measures the effects of these bioactive compounds on the mineral status, BBM morphology or functionality.

In the present study, we evaluated the effects of the intra-amniotic administration of resveratrol and pterostilbene on the relevant physiological, molecular and microbial parameters in vivo (*Gallus gallus*). Furthermore, the concentrations of the two stilbenes were determined in the red grape cultivars of three different species grown in the New York State Finger Lakes region: *Vitis vinifera*, *Vitis labruscana* and a French hybrid. This was done in order to associate the findings of this study with the practical dietary health benefits of grape and grape product consumption. Similarly, the synergistic group concentration was selected based on the realistic doses of resveratrol and pterostilbene found in nature [50].

Previous studies have shown that the intra-amniotic administration of prebiotics, soluble fiber and polyphenols affects the BBM morphology, specifically the villus surface area and goblet cell number, type and size [19,20,23,24,25,26]. In this study, the results indicate a significant increase in the duodenal villus length and diameter that was observed in all stilbenes treatment groups (5% resveratrol, 5% pterostilbene and synergistic). Furthermore, a significant increase in the goblet cell diameter was measured in the stilbene treatment groups relative to the controls (no injection, 18 MΩ H_2_O and 5% inulin), which suggests an increased capacity for mucus production in the intestinal lumen. Goblet cells are mucus producing and secreting cells [51]. The mucin produced has the essential function of housing certain intestinal microbial populations [52] and contribute to nutrient digestion and absorption [53]. Changes in intestinal morphology are associated with the improvement in mineral status through enhanced bacterial composition and function in the gut. This observation demonstrates that stilbene administration induced enterocyte cell proliferation, which is considered a mechanical indicator of the BBM morphology, functionality and digestive and absorptive capabilities [28].

In the context of mineral absorption, as previously demonstrated [23,25,26,27,28,54], the bacterial fermentation activity led to increased production of short-chain fatty acids (SCFAs), which may indirectly result in increased mineral absorption. In the present study, there was downregulation in the relative expression of DcytB (Fe reductase) in all treatment groups compared with the controls (Figure 1), this can suggest improved Fe bioavailability. To support this, we observed higher serum Fe and Zn concentrations and higher Zn storage in the liver samples of the synergistic group (Table 3), indicating that stilbene treatments have potential to improve the Fe and Zn metabolism-related pathways, and more studies are needed to confirm this observation. The low hemoglobin and high serum Fe concentrations observed in the synergistic group could be explained by the presence of more unbound serum Fe versus bound hemoglobin or Fe. However, a long-term feeding trial is warranted to further elucidate the possible mechanism of these effects. This is a novel finding that demonstrates improved mineral status and digestive capabilities in vivo. In accordance with this result, we observed upregulation in the gene expression of Δ-6-desaturase, a Zn-dependent enzyme, in the stilbene treatment groups. This observation suggests that the Zn’s physiological status was improved and that Zn was present in a sufficient amount to support the appropriate function of this Zn-dependent enzyme [40,55].

In addition, upregulation in the gene expression of proteins involved with calcium and magnesium metabolism in the 5% pterostilbene and synergistic groups, relative to the 5% inulin control, was detected. This observation agrees with our hypothesis that the intra-amniotic administration of stilbenes beneficially modulates mineral absorption and therefore may improve BBM functionality.

The BBM functionality was appraised by measuring the gene expression of the functional proteins as biomarkers of BBM digestive and absorptive capabilities. The SGLT1 gene expression was upregulated in the stilbene treatment groups compared with the controls (Figure 2). SGLT1 is a sodium-glucose transport protein responsible for dietary glucose absorption [56], and in agreement with our results, other studies have shown an increase in the gene expression of SGLT1 with the intra-amniotic administration of prebiotic-capacity soluble extracts [19,23]. In addition, it was previously suggested that SGLT1 activity contributes to the upregulation of the gene expression of intestinal mucin producing proteins via activation of a protein kinase C-dependent pathway that is controlled by the glucose uptake [57,58]. This mechanism can explain the increased goblet cell diameter in the stilbene treatment groups and further supports the improvement in BBM functionality.

Despite the low bioavailability of polyphenolic compounds in the gastrointestinal tract [59,60], stilbenes showed anti-inflammatory activity by the downregulation of IL-1β and TNF-α gene expressions compared with the inulin control (Figure 2). Comparable results were demonstrated in vivo [61] and in a study with humans [62], suggesting the potent action of these compounds on anti-inflammatory pathways. However, despite the antihypertensive activity demonstrated by stilbenes [42,63,64], we did not observe differences in the gene expressions of ACE and AT1R between the experimental groups. This may be due to acute exposure [65].

An additional objective of this study was to evaluate the specific effect of the stilbene treatments on the intestinal microbiome. A bidirectional relationship exists between the host and microbes that is vital to intestinal functionality and overall health [6,66,67]. We hypothesized that the stilbene treatments would alter the composition and function of the gut microbiome, similar to what previous studies found [49,68]. In the current study, the microbial analyses indicated that the 5% inulin treatment group differed in the distance metrics compared with the other treatment groups (Figure 3B). The β diversity measured by Jaccard distances revealed that the treatment groups with stilbenes displayed strong clustering. Furthermore, the strong clustering between the 5% resveratrol and synergistic groups (Figure 3C) indicated that their bacterial compositions were similar, which is unsurprising since these groups were compositionally similar. (The synergistic group received 47.5 mg mL^−1^ of resveratrol and 2.5 mg mL^−1^ of pterostilbene, and the resveratrol group received 50 mg mL^−1^ of resveratrol) In addition, the microbial α diversity showed a lower bacterial abundance in the synergistic treatment group (Figure 3A), which is an interesting result that merits future investigation.

Furthermore, we observed a lower abundance (<0.5%) of Actinobacteria in the synergistic group compared with the 5% inulin control. In addition, the trend observed in the synergistic group suggests a decreased abundance of Firmicutes (*p* = 0.083). This could be considered a positive result, as the Firmicutes phylum is associated with being overweight and obesity in children and adults [69]. Previously, the consumption of resveratrol was shown to reduce the abundance of Firmicutes [70]. Certain members of this phylum produce trimethylamine-N-oxide (TMAO) as metabolites, which was recently linked to cardiovascular disease pathogenesis [71]. Since Firmicutes partially modulate the catabolism of choline and carnitine to TMAO [71,72], the suggested reduction in this phylum might be beneficial to the host.

Furthermore, a general taxonomic difference using LEfSe was observed between the 5% pterostilbene group and the no injection control (Figure 5B), whereby the Bacteroidetes predominated in the 5% pterostilbene group. Bacteroidetes are SCFA-producing bacteria and mostly produce propionate and succinate [73]. We also observed that the abundance of the S24-7 family and *Prevotella* genus, belonging to the Bacteroidetes phylum, were enriched. Members of the S24-7 family, currently named Muribaculaceae [74], can ferment polysaccharides into SCFAs. We also observed that the Clostridiales order was enriched in the 5% pterostilbene group. This observation agrees with a previous clinical study by Calderón-Pérez et al. [75] which recently demonstrated a positive correlation (*p* = 0.037) between stilbenes and the abundance of the Clostridiales order. The elevation in SCFA-producing bacterial populations suggests an increased SCFA concentration in the lumen. As previously demonstrated, these SCFAs may lead to intestinal cellular (specifically enterocyte) proliferation [76]. This connection could explain the increase in the duodenal villi length, villi diameter and goblet cell diameter (Table 4 and Table 5) compared with the controls. Furthermore, by favoring bacterial fermentation, stilbenes affected the intestinal luminal pH, acidifying it and improving mineral (as Fe and Zn) solubility [17,19,21]. This could tie together with the improved Fe and Zn mineral status observed in this study (Table 3).

In addition, the gut microbiome data demonstrates how the detected and specific microbial profile within a treatment group is linked to the host’s physiological status. The results show that the 5% pterostilbene group upregulated the pathways associated with cellular processes (e.g., DNA repair and recombination proteins, cell division, DNA replication and fatty acid biosynthesis) and metabolic processes (e.g., amino acid, sugar and nucleotide sugar metabolism, energy metabolism and glycolysis or gluconeogenesis metabolism) (Figure 6). We observed a significant difference in the KEGG microbial metabolic pathway analysis between the 5% pterostilbene group and all the other groups. This could be explained by the fact that pterostilbenes are highly absorbable (about 80%) or bioavailable when compared with resveratrol (about 20%) [11,12]. Additionally, the antioxidant and anti-inflammatory properties of stilbenes could beneficially modulate the gut microbiome [24], leading to the observed results.

## 5. Conclusions

The present study is the first to demonstrate changes in the duodenal morphometric parameters after the intra-amniotic administration of stilbenes, which have been shown to improve micronutrient absorption. The 5% pterostilbene and synergistic treatments (4.75% resveratrol and 0.25% pterostilbene) improved BBM functionality, and as a result, led to an increase in the Fe and Zn serum concentrations. Although we did not detect significant modifications in the taxonomy of the cecal microbiota, upregulation in the SCFA-producing bacteria in the 5% pterostilbene group was observed. This modulation affected several bacterial metabolic pathways related to bacterial, cellular and metabolic processes. This study presents promising evidence to encourage dietary stilbene consumption. However, future long-term studies are necessary to further evaluate the nutritional benefits of dietary stilbenes.

## Figures and Tables

**Table 1:** Sequences of experimental primers.

Analyte	Forward Primer (5′-3′)	Reverse Primer (5′-3′)	Base Pairs Length	GI Identifier
*Calcium Metabolism*				
TRPV6	GCTCCCAGAACCTTCTCTATTT	CCAGGTAATCCTGAGCTCTAATG	123	418,307
PMCA1b	TGCAGATGCTGTGGGTAAAT	CCATAAGGCTTCCGCAATAGA	100	374,244
NCX1	CCTGACGGAGAAATAAGGAAGA	CCCAGGAGAAGACACAGATAAA	114	395,760
*Iron Metabolism*				
DMT1	TTGATTCAGAGCCTCCCATTAG	GCGAGGAGTAGGCTTGTATTT	101	206,597,489
Ferroportin	CTCAGCAATCACTGGCATCA	ACTGGGCAACTCCAGAAATAAG	98	61,098,365
DcytB	CATGTGCATTCTCTTCCAAAGTC	CTCCTTGGTGACCGCATTAT	103	20,380,692
*Immune Response*				
IL-1β	CTCACAGTCCTTCGACATCTTC	TGTTGAGCCTCACTTTCTGG	119	88,702,685
IL-6	ACCTCATCCTCCGAGACTTTA	GCACTGAAACTCCTGGTCTT	105	302,315,692
TNF-α	GACAGCCTATGCCAACAAGTA	TTACAGGAAGGGCAACTCATC	109	53,854,909
*Magnesium Metabolism*				
MRS2	GCTGGTAACCGGGATTATGT	GCAGGAACATGAGGAGGTAAT	105	420,820
TRPM6	ACAGATGCTGCTGACTGATATG	AAGATAGTGGGTGGTAGGAGAA	99	1,008,596,03
TRPM7	GCGTGGGATAGAGTTGACATT	TCACAAGGGCATCCAACATAG	100	427,502
*Zinc Metabolism*				
ZnT1	GGTAACAGAGCTGCCTTAACT	GGTAACAGAGCTGCCTTAACT	105	54,109,718
ZnT7	GGAAGATGTCAGGATGGTTCA	CGAAGGACAAATTGAGGCAAAG	87	56,555,152
ZIP9	CTAAGCAAGAGCAGCAAAGAAG	CATGAACTGTGGCAACGTAAAG	100	237,874,618
Δ-6-desaturase	GGCGAAAGTCAGCCTATTGA	AGGTGGGAAGATGAGGAAGA	93	261,865,208
*Hypertension*				
ACE *	CATGGCCTTGTCTGTCTCC	GAGGTATCCAAAGGGCAGG	142	424,059
AT1R *	TCATCTGGCTCCTTGCTGG	AACCTAGCCCAACCCTCAG	138	396,065
*BBM Functionality*				
AP	CGTCAGCCAGTTTGACTATGTA	CTCTCAAAGAAGCTGAGGATGG	138	45,382,360
SI	CCAGCAATGCCAGCATATTG	CGGTTTCTCCTTACCACTTCTT	95	2,246,388
SGLT1	GCATCCTTACTCTGTGGTACTG	TATCCGCACATCACACATCC	106	8,346,783
18s rRNA	GCAAGACGAACTAAAGCGAAAG	TCGGAACTACGACGGTATCT	100	7,262,899

TRPV6: transient receptor potential cation channel subfamily V member 6; PMCA1b: plasma membrane Ca^2+^ATPase isoform 1b; NCX1: sodium-calcium exchanger 1; DMT1: divalent metal transporter 1; Dcytb: duodenal cytochrome b; IL1β: interleukin 1 beta; IL6: interleukin 6; TNF-α: tumor necrosis factor alpha; MRS2: mitochondrial magnesium channel type 2; TRPM6 and TRPM7: transient receptor potential melastatin; ZnT and ZIP: zinc transporter proteins; ACE: angiotensin-converting enzyme; AT1R: angiotensin II receptor type I; AP: amino peptidase; SI: sucrose isomaltase; SGLT1: sodium-glucose transport protein 1; 18S rRNA: 18S ribosomal subunit. * Heart analysis.

**Table 2:** Polyphenols present within 3 grape varieties.

Polyphenolic Compounds	Mean (Da)	M + H (Da)	Retention Time (min)	Concentration (ng/g)
Resveratrol	228.24	229.24	5.334 ± 0.008	CF: 64.7 ± 3.9 Noire: 60.2 ± 2.8 Concord: 1.85 ± 0.3
Pterostilbene	256.3	257.3	10.341 ± 0.007	ND

Values are the means ± standard deviation (*n* = 7). Da: daltons; min: minutes; M + H: mass + hydrogen data; CF: Cabernet Franc grape variety; ND: not determined.

**Table 3:** Blood hemoglobin (Hb) concentration and Fe and Zn contents.

Treatment Group		Liver (µg/g)		Serum (µg/g)	
	Hb (g/dL)	Fe	Zn	Fe	Zn
No Injection	8.75 ± 0.80 ^a^	32.47 ± 2.83 ^b^	15.79 ± 0.95 ^b^	2.09 ± 0.24 ^b^	0.86 ± 0.08 ^c^
18 MΩ H_2_O	8.70 ± 0.59 ^a^	37.93 ± 4.93 ^a,b^	16.12 ± 0.96 ^b^	2.00 ± 0.27 ^b^	0.84 ± 0.07 ^c^
5% Inulin	8.86 ± 0.69 ^a^	48.96 ± 4.39 ^a^	18.23 ± 0.88 ^a,b^	2.76 ± 0.33 ^b^	0.97 ± 0.08 ^b,c^
5% Resveratrol	9.71 ± 0.53 ^a^	46.45 ± 4.71 ^a^	16.72 ± 1.58 ^b^	2.42 ± 0.23 ^b^	0.97 ± 0.12 ^b,c^
5% Pterostilbene	8.95 ± 0.77 ^a^	37.43 ± 1.52 ^a,b^	14.43 ± 0.49 ^b^	2.94 ± 0.44 ^b^	1.33 ± 0.20 ^a^
Synergistic	8.04 ± 0.94 ^b^	50.96 ± 6.08 ^a^	21.22 ± 3.36 ^a^	3.87 ± 0.41 ^a^	1.24 ± 0.11 ^a,b^

Values are the means ± SEM (*n* = 8). ^a–c^ Treatment groups not indicated by the same letter are significantly different (*p* < 0.05). Synergistic: 4.75% resveratrol, 0.25% pterostilbene.

**Table 4:** Effect of the intra-amniotic administration of stilbenes on the duodenal small intestinal villi length, villi diameter and crypt depth.

Treatment Group	Villus Length (µm)	Villus Diameter (µm)	Depth of Crypts (µm)
No Injection	235.86 ± 4.08 ^e^	52.14 ± 0.67 ^b^	66.30 ± 1.33 ^a^
18 MΩ H_2_O	223.29 ± 4.53 ^e^	52.52 ± 5.21 ^c^	53.42 ± 1.11 ^b^
5% Inulin	262.54 ± 3.76 ^d^	44.57 ± 0.79 ^d^	51.30 ± 1.07 ^b^
5% Resveratrol	294.92 ± 3.48 ^c^	54.47 ± 0.70 ^b^	63.73 ± 1.20 ^a^
5% Pterostilbene	451.58 ± 11.2 ^b^	81.33 ± 4.95 ^a^	66.41 ± 1.19 ^a^
Synergistic	573.92 ± 9.14 ^a^	77.37 ± 0.99 ^a^	63.98 ± 1.13 ^a^

Values are the means ± SEM (*n* = 5). ^a–e^ Treatment groups not indicated by the same letter are significantly different (*p* < 0.05).

**Table 5:** Effect of the intra-amniotic administration of stilbenes on the goblet cells of the small intestine.

Treatment Group	Goblet Cell Diameter (µM)	Crypt Goblet Cell Number	Villus Goblet Cell Type Number		
			Acidic	Neutral	Mixed
No Injection	7.27 ± 0.05 ^c^	9.53 ± 0.32 ^c,d^	10.22 ± 0.26 ^b^	0.12 ± 0.03 ^a^	0.13 ± 0.03 ^c,d^
18 MΩ H_2_O	6.75 ± 0.05 ^e^	10.01 ± 0.22 ^c^	9.46 ± 0.23 ^c^	0.00 ± 0.00 ^b^	0.05 ± 0.03 ^d,e^
5% Inulin	7.12 ± 0.06 ^d^	12.27 ± 0.33 ^a^	10.65 ± 0.25 ^a,b^	0.00 ± 0.00 ^b^	0.02 ± 0.01 ^e^
5% Resveratrol	7.94 ± 0.06 ^b^	10.91 ± 0.21 ^b^	10.97 ± 0.23 ^a^	0.00 ± 0.00 ^b^	0.17 ± 0.03 ^c^
5% Pterostilbene	7.81 ± 0.07 ^b^	10.04 ± 0.18 ^c^	10.78 ± 0.22 ^a,b^	0.00 ± 0.00 ^b^	0.28 ± 0.05 ^b^
Synergistic	8.55 ± 0.07 ^a^	9.02 ± 0.15 ^d^	8.29 ± 0.17 ^d^	0.00 ± 0.00 ^b^	0.66 ± 0.05 ^a^

Values are the means ± SEM (*n* = 5). ^a–e^ Treatment groups not indicated by the same letter are significantly different (*p* < 0.05).
